# Development of a Novel *In Silico* Docking Simulation Model for the Fine HIV-1 Cytotoxic T Lymphocyte Epitope Mapping

**DOI:** 10.1371/journal.pone.0041703

**Published:** 2012-07-27

**Authors:** Masahiko Mori, Kei Matsuki, Tomoyuki Maekawa, Mari Tanaka, Busarawan Sriwanthana, Masaru Yokoyama, Koya Ariyoshi

**Affiliations:** 1 Institute of Tropical Medicine, Nagasaki University, Nagasaki City, Nagasaki, Japan; 2 Department of Paediatrics, The Peter Medawar Building for Pathogen Research, University of Oxford, Oxford, United Kingdom; 3 Department of Medical Sciences, Ministry of Public Health, Nonthaburi, Thailand; 4 Pathogen Genomics Center, National Institute of Infectious Diseases, Shinjuku-ku, Tokyo, Japan; University of Alabama at Birmingham, United States of America

## Abstract

**Introduction:**

Class I HLA's polymorphism has hampered CTL epitope mapping with laborious experiments. Objectives are 1) to evaluate the novel *in silico* model in predicting previously reported epitopes in comparison with existing program, and 2) to apply the model to predict optimal epitopes with HLA using experimental results.

**Materials and Methods:**

We have developed a novel *in silico* epitope prediction method, based on HLA crystal structure and a peptide docking simulation model, calculating the peptide-HLA binding affinity at four amino acid residues in each terminal. It was applied to predict 52 HIV best–defined CTL epitopes from 15-mer overlapping peptides, and its predictive ability was compared with the HLA binding motif-based program of HLArestrictor. It was then used to predict HIV-1 Gag optimal epitopes from previous ELISpot results.

**Results:**

43/52 (82.7%) epitopes were detected by the novel model, whereas 37 (71.2%) by HLArestrictor. We also found a significant reduction in epitope detection rates for longer epitopes in HLArestrictor (p = 0.027), but not in the novel model. Improved epitope prediction was also found by introducing both models, especially in specificity (p<0.001). Eight peptides were predicted as novel, immunodominant epitopes in both models.

**Discussion:**

This novel model can predict optimal CTL epitopes, which were not detected by an existing program. This model is potentially useful not only for narrowing down optimal epitopes, but predicting rare HLA alleles with less information. By introducing different principal models, epitope prediction will be more precise.

## Introduction

Cytotoxic T lymphocytes (CTLs) play a crucial role in HIV replication control by eliminating virus-infected cells by recognizing class I Human Leukocyte Antigen (HLA) molecule-viral peptides ( = epitope) complex. This response is thought to be a major determinant of the viral set point, and consequent disease progression [1]. However the efficacy of the CTL response is affected by the extent of polymorphisms in HLA loci and viral sequences. The HLA region is found on chromosome 6 and is the most polymorphic loci in the human genome [2]; each individual expresses up to six different class I alleles out of a vast pool of allelic variants, the reported number of which reaches 5,399 for class I HLA molecules (1,757 of HLA-A, 2,338 of HLA-B, and 1,304 of HLA-C alleles) [3]. In addition, the extensive diversity of HIV-1 owing to its extreme capacity to mutate has led to a reported 13 prototype clades and 43 circulating recombinant forms (CRFs) [4]. Despite such HLA polymorphism and HIV viral diversity environment, recent genome wide association study (GWAS) reported the best contribution of class I HLA for viral control, suggesting the importance of CTL epitope mapping with responsible HLA information [5]. Several major HIV-1 epitopes and their restricting HLA alleles have been defined through fine epitope mapping; 1,344 epitopes and their restricting HLA alleles have been reported as of February 2012 (CTL Epitopes. Los Alamos National Lab. http://www.hiv.lanl.gov/). The limitation of the dataset currently available however, is that the majority of these epitope/HLA combinations are derived from subtype B-infected Caucasians or C-infected Africans, and epitope information from other subtypes or ethnicities is rare.

The traditional, *in vitro* method of epitope detection involves using a matrix of overlapping peptides (OLPs) encoding viral proteins in Enzyme-Linked Immunospot (ELISpot) assays to identify a single candidate peptide, from which the 8-11mer epitope is mapped down. This is typically followed by the confirmation of the restricting HLA alleles using tetramers or in a ^51^Cr release assay using peptide-specific lines [6], [7]. It is a difficult and labor-intensive process, particularly time-consuming in the case of epitopes restricted by rare HLA alleles because of the limited number of samples available.

Recently, alternative, *in silico* models for epitope prediction have been developed [8]. These can broadly be divided into two models; the first is an algorithm based on the peptide-binding motif, and the second is a structural algorithm model based on the crystal structure of HLA molecules. The former is characterized by the use of motif matrices deduced from refined motifs based on the pool sequence, enlisting optimal amino acid sequences at anchor positions in specific HLA alleles. An example of such an algorithm is the SYFPEITHI [9] database, which predicts the HLA-binding affinities of peptides by ranking them according to the presence of primary and secondary anchor amino acids. However these models are based on reported epitopes and their restricting HLA alleles, so their predictions are powerful in the context of well-published HLA alleles but not suitable against rare or novel alleles with little previous information. Another model of epitope prediction is the binding affinity model, which calculates the peptides' binding affinity and scores it using quantitative matrices (QMs), a well-known example being the NetMHC [10], [11] or the HLArestrictor [12]. This model scores binding strength as binding affinity with thresholds to differentiate strong binding peptides and weak ones in each calculation.

On the other hand, the structural algorithm model does not require binding motif information, which is advantageous for the definition of epitopes restricted by HLA alleles with less published epitope information. Recently, a docking simulation model (DSM) which takes into consideration binding energy such as electrostatic interactions and van der Waals (vdw) interactions, together with the crystal structure of HLA alleles, has been developed [13]–[17].

Our objectives here are 1) to evaluate the novel *in silico* DSM in predicting previously reported best-defined epitopes in comparison with existing binding motif-based program, and 2) to apply the model to predict optimal size of the epitopes and restricting HLA alleles using results obtained from our previous study in a HIV-1 CRF01_AE-infected Thai cohort.

## Materials and Methods

### Ethic Statement

This study was approved by Thai Ministry of Public Health Ethics Committee. Written informed consent was obtained from all patients after explaining the purpose and expected consequences of the study.

### Computational program and calculation

We used the commercial softwares Molecular Operating Environment® (MOE) (CCG Inc., Montreal, Canada) and MOE-ASEDock® (Ryoka System Inc., Tokyo, Japan) for the molecular binding affinity calculation [18]. HLA's 3D models were obtained from the X-ray crystallography database in MOE's library (1OGA for HLA-A*02:01, IQ94 for HLA-A*11:01, 2BCK for HLA-A*24:02, 1XR9 for HLA-B*15:01, 1JGE for HLA-B*27:05, 2CIK for HLA-B*35:01, 1E27 for HLA-B*52:01, 2RFX for HLA-B*57:01, and 1EFX for HLA-C*03:04). In cases where the original X-ray crystallography information was unavailable, we generated a 3D structural model using highly homologous HLA alleles as template, using rotamer explorer or homology modeling to reconstruct their structures by changing sequential difference sites, a method originally used in the point mutation program attached in MOE AMBER99 [19] for force field, calculations. For solvent effect energy calculation, a generalized Born model [20], were introduced. As an indicator of the affinity between epitope candidate peptides and the class I HLA allele, we measured the U_dock score [U_ele (electric energy)+U_vdw (van der Waals energy)+U_solv (Solvation energy)+U_strain (Strain energy)] (kcal/mol) [18]. We calculated the U_dock score of four residues at each N- and C-terminal, spanning the anchor position at each of the terminals, and scored the sum of them as binding affinity. A lower score indicates a higher affinity between the HLA molecule and peptides.

### Evaluation of the novel DSM through an analysis of best-defined HIV CTL epitopes and their restricting HLA alleles

**Figure 1:**
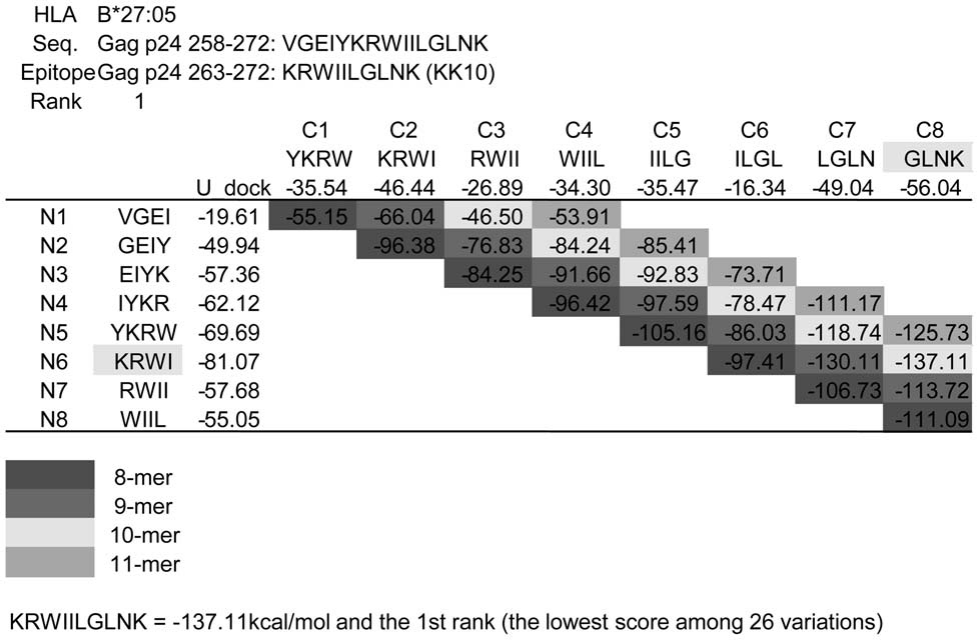
Example of epitope prediction using the novel *in silico* docking simulation model. U_dock scores of the N-terminal (Row N1–N8) and C-terminal (Column C1–C8) was calculated and their sum was scored as the U_dock score (kcal/mol) of each 8 to 11-mer peptide's. The lower score indicated stronger binding between the peptide and HLA. In this example, Gag p24_263–272_ KRWIILGLNK (KK10), well-known as one of the best-defined epitopes, scored −137.11 kcal/mol against HLA-B*27:05 and was the lowest (ranked as the 1st) among 26 variants in 15-mer peptide of Gag p24_258–272_ VGEIYKRWIILGLNK.

For the quality evaluation of this novel program, we first calculated the U_dock score for 52 best-defined HIV epitopes restricted by the alleles HLA-A*02:01, HLA-A*11:01, HLA-A*24:02, HLA-B*15:01, HLA-B*27:05, HLA-B*35:01 and HLA-B*57:01 as enlisted in Los Alamos database (CTL Epitopes. Los Alamos National Lab. http://www.hiv.lanl.gov/). We calculated the U_dock score between the restricting HLA alleles and the 8 to 11-mer peptides within 15-mer peptides of the viral strain HXB2, in which best-defined epitopes were included. 26 variants of 8 to 11-mer peptides were calculated in one HLA and 15-mer peptide combination, then the lowest U_dock score was ranked as the 1st and the highest score as the 26th in each calculation (Figure 1). Combinations that ranked within the top five were regarded as positive. In parallel with our DSM, we also performed epitope prediction using the latest artificial neural network (ANN) model, the HLArestrictor [12], using the affinity thresholds of Strong Binder (SB), Weak Binder (WB), Combined Binder (CB) and Non-binder (NB), according to their definitions.

We evaluated the sensitivity, specificity, positive predictive value (PPV), and negative predictive value (NPV) for each best-defined epitope prediction using the DSM, HLArestrictor, as well as those defined as dual positive by both models.

### Analysis of *in vitro* HIV-1 CRF01_AE Gag epitope candidates by using both *in silico* epitope prediction models

We then applied both the DSM and the HLArestrictor to predict the optimal size of epitopes, based on results obtained from our previous study [21], in which 31 candidate epitopes were detected by ELISpot assays using Gag 15-mer OLPs and their HLA associations detected by Fisher's exact test in a cohort of 137 (107 female and 30 male) HIV-1 CRF01_AE-infected Thais. All were chronically infected and treatment naïve, with median 461/ul CD4+T cell count (range 204–1,191) and 4.2 log copies/ml viral load (2.6–5.9).

### Epitope prediction for the immunogenic Gag OLP p24_276–285_ MYSPVSILDI using a ^51^Cr release assay and both *in silico* models

In our previous study [21], the 15-mer peptides Gag p24_271–285_ NKIVRMYSPVSILDI (NI15) and p24_276–290_ MYSPVSILDIRQGPK (MK15) induced the largest responses in terms of both breadth and magnitude, and were statistically associated with the alleles HLA-A*02:07, HLA-B*46:01, and HLA-C*01:02, which were under linkage disequilibrium (LD) association [21]. Presuming that the optimal epitope resides in the overlapping amino acid sequence between NI15 and MK15, that is, p24_276–285_ MYSPVSILDI (MI10), we conducted a ^51^Cr release assay as previously described [22].

## Results

### Prediction of best-defined epitopes by the DSM and the peptide binding motif model

We have evaluated the predictive power of our DSM by testing its ability to predict epitopes within 52 15-mer peptides spanning the epitopes for seven HLA alleles enlisted in the Los Alamos database as best-defined epitopes. Overall, DSM ranked 43/52 (82.7%) of the best-defined epitopes correctly within the top five candidates, within which 14 epitopes ranked as the 1st, 11 as the 2nd, 7 as the 3rd, 3 as the 4th, then 8 as the 5th (Table S1). This was comparable to the HLArestrictor, where 37/52 (71.2%, 43/52 vs 37/52, p = 0.24 by Fisher's exact test) best-defined epitopes scored within the threshold of binding affinity without having 4 or more other candidate epitopes: 20 as SB, 10 as WB and 7 as CB. Table 1 summarizes the performance on epitope prediction by each model and dual positives by both models, according to their sensitivity, specificity, PPV and NPV. The performance of the DSM is similar to that of HLArestrictor. Interestingly, by introducing both models, specificity increased with significance (p<0.001), and an additive effect was seen in the PPV. We believe this is the first study to report a structure-based epitope prediction model with comparable or greater predictive power than a peptide-binding motif based model.

**Table 1:** Evaluation of best-defined epitope prediction among docking simulation model, HLArestrictor, and positives in dual models.

	DSM	HLArestrictor	Dual positives	p (mxn Fisher's exact test)
Sensitivity	0.83	0.71	0.62	0.056
Specificity	0.83	0.94	0.97	<0.001
PPV	0.17	0.31	0.43	0.095
NPV	0.99	0.99	0.98	0.46

Evaluation of best-defined epitope prediction among each model and positives in dual models were statistically evaluated, according to their sensitivity, specificity, positive prediction value (PPV) and negative prediction value (NPV) by maximum Fisher's exact test. DSM: Docking simulation model.

32/52 (61.5%) epitopes were detected as a significant epitope candidate by both models. 11/52 (21.2%) epitopes were detected only by the DSM, while 5/52 (9.6%) were detected only by HLArestrictor. 4/52 (7.7%) epitopes were not detected by either methods. Within the 14 epitopes not correctly predicted by HLArestrictor, incorrect epitopes were predicted in 7 epitopes. It is noteworthy that two epitopes, Nef_75–82_ PLRPMTYK (PK8) restricted by HLA-A*11:01 and Nef_117–127_ TQGYFPDWQNY (TY11) restricted by HLA-B*15:01 were detected as a NB by HLArestrictor, whereas they were ranked as the 2nd in PK8 and the 1st in TY11 in the DSM. Integrase_179–188_ AVFIHNFKRK (AK10) restricted by HLA-A*11:01 was predicted as a SB, but because there were 5 other SB candidates, 3 WB candidates and 1 CB candidate, this prediction was regarded as failure.

A striking feature of the DSM was that it had a high detection rate of best-defined epitopes independent of the peptide's length. The prediction rate of shorter epitopes (8 and 9-mer) was 27/31 (87.1%) while the rate for longer epitopes (10 and 11-mer) was 16/21 (76.2%), between which we found no significant difference by Fisher's exact test (p = 0.46). In contrast, the ability of HLArestrictor to accurately predict best-defined epitopes was highly dependent on epitope length, as the prediction rate of longer epitopes (11/21, 52.3%) was significantly lower than that of shorter ones (26/31, 83.9%) (p = 0.027).

Successful prediction with the DSM was dependent on the HLA allele and its peptides: in HLA-B*15:01, HLA-B*27:05 and HLA-B*35:01, all of the best-defined epitopes were ranked within the top 5th. However, four best-defined epitopes restricted by HLA-B*57:01 and HLA-A*02:01 scored within the worst 5th candidates: Nef_120–128_ YFPDWQNYT, p15_433–442_ FLGKIWPSYK, RT_33–41_ ALVEICTEM, and p24_161–172_ KAFSPEVIPMF.

### Optimal epitope prediction to analyze HIV-1 CRF01_AE Gag ELISpot assay data using two *in silico* models

We next applied the model to predict optimal epitopes against HIV-1 CRF01_AE Gag based on our previously obtained results in a Thai HIV cohort study [21]. In total, 31 peptide-HLA associations were analyzed: 5 in HLA-A, 13 in HLA-B, and 13 in HLA-C (Table S2). Among these, 10 overlapping peptides spanned previously reported epitopes (6 were best-defined epitopes and 4 were published but not enlisted as best-defined epitopes). In the DSM, 9/10 (90%) reported epitopes were successfully ranked within the 5th as significant epitope candidates, and all of the six best-defined epitopes ranked either the 1st or 2nd. In HLArestrictor, 8/10 (80%) epitopes were predicted as significant binders; 3 as SB, 4 as WB, and 1 as CB, but 2 epitopes (best-defined epitopes HLA-A*02:07-restircted YL9, and HLA-B15-restricted KL9) were not predicted as significant binders. HLArestrictor also predicted another 16 sequences as potential epitope candidates: 1 as SB, 12 as WB, and 2 as CB. Intriguingly only one WB candidate was ranked within the top five by the DSM, reflecting a considerable degree of discrepancy between the two prediction methods.

8 previously unreported peptides were predicted by both models: HLA-B*38:02-restricted p24_198–205_ MQMLKETI (rank 1st in DSM and WB in HLArestrictor), HLA-B*40:01-restricted p24_311–321_ QEVKNWMTETL (2nd and SB), HLA-B*46:01-restricted p24_275–283_ RMYSPVVSIL (5th and SB), HLA-B*58:01-restircted p17_79–86_ YNTVVTLW (1st and WB), HLA-B*58:01-restricted p17_77–86_ SLYNTVVTLW (4th and WB), HLA-C*01:02-restricted p24_277–285_ YSPVSILDI (2nd and WB in p24_271–285_ and 3rd and WB in p24_276–290_), HLA-C*01:02-restricted p24_276–285_ MYSPVSILDI (4th and WB both in p24_271–285_ and p24_276–290_), and HLA-C*01:02-restricted p24_296–304_ YVDRFYKTL (1st and WB).

### Application of the *in silico* DSM to define the restricting HLA molecule

We conducted a ^51^Cr release assay with a truncated peptide titration spanning the overlapping region between Gag p24_271–285_ NKIVRMYSPVSILDI (NI15) and p24_276–290_ MYSPVSILDIRQGPK (MK15). These induced the largest responses both in breadth and magnitude in our previous study, and were statistically associated with HLA-A*02:07, HLA-B*46:01, and HLA-C*01:02, which we calculated to be under LD association [21]. We found strong killing against HLA-B*46:01 and HLA-C*01:02-matched p24_276–285_ MYSPVSILDI (MI10)- and p24_277–285_ YSPVSILDI (YI9)-pulsed target cells but not in any other condition (Figure S1). However, we could not further specify the restricting HLA molecule because a single HLA-matched target cell was not available due to the strong LD between them. Therefore, we conducted *in silico* analysis in order to identify the responsible HLA. Table 2 shows the results of the DSM between these two peptides (MI10 and YI9) and three candidate HLA alleles (HLA-A*02:07, HLA-B*46:01 and HLA-C*01:02). Firstly, with the DSM, none of these two peptides were predicted within the top five candidate epitopes when binding to HLA-A*02:07 or HLA-B*46:01, and neither scored significant binding using the HLArestrictor, eliminating these as the restricting HLA molecules. However in the model with HLA-C*01:02, both two peptides ranked within the 5th; MI10 ranked as the 3rd in NI15 and the 4th in MK15, while YI9 was ranked as the 2nd in NI15 and the 3rd in MK15. Significant binding affinity of MI10 and YI9 to HLA-C*01:02 was also predicted by HLArestrictor. Secondly, in the binding motif of HLA-C*01:02 (x[AL][P]xxxxx[L]), both MI10 and YI9 encoded compatible or similar hydrophobic amino acids with the binding motif x[Y]xxxxxxx[I] in MI10 and xx[P]xxxxx[I] in YI9. Together, these results indicate that the optimal epitopes MI10 and YI9 are equally likely candidates recognized by HLA-C*01:02, with YI9 ranking slightly higher in the DSM.

**Table 2:** Prediction of the HLA restriction of Gag p24_276–285_ MYSPVSILDI (MI10) and p24_277–285_ YSPVSILDI (YI9) using *in silico* methods.

			U_dock rank		
HLA	Binding motif	Peptide	NI15	MK15	HLArestrictor
A*02:07	x[L][D]xxxxx[L]	MI10	13	13	
		YI9	14	16	
B*46:01	x[M(I)]xxxxxx[YF]	MI10	15	20	
		YI9	19	21	
C*01:02	x[AL][P]xxxxxx[L]	MI10	3	4	WB
		YI9	2	3	SB

HLA restriction prediction against two reactive Gag peptides, Gag p24_276–285_ MYSPVSILDI (MI10) and p24_277–285_ YSPVSILDI (YI9) was performed by the docking simulation model, and the binding motif HLArestrictor 1.2. The U_dock rank by the docking simulation model against MI10 and YI9 was analyzed in the original 15-mer peptides of Gag p24_271–285_ NKIVRMYSPVSILDI (NI15) and p24_276–290_ MYSPVSILDIRQGPK (MK15). SB: Strong Binder, WB: Weak Binder.

## Discussion

In this study, we demonstrated that the structure-based DSM can predict the peptide binding affinity with various HLA molecules, independently of peptide binding motif information. To our knowledge, this novel DSM is the first model of its kind that succeeded in predicting HIV-1 CTL best-defined epitopes, with better or at least equivalent accuracy to the latest binding motif-based program. We also found a high detection rate of best-defined epitopes independent of peptide size in the DSM, while the detection rate significantly decreased with longer epitopes in the other model.

Historically, comparisons of epitope prediction methods has generally shown that peptide-binding motif based methods outperform structure-based methods [23]. However, the increased availability of crystal structures of MHC-peptide complexes is enabling the development of prediction methods using such structural models and the calculation of free energy of binding [23], [24]. In the review by Liao *et al* [23], their comprehensive comparison of structure-based models and peptide-binding motif models in epitope prediction showed that the structure-based model was able to outperform all other methods except the ANN model, which performed equally well. In our novel program, we use a measure of the binding affinity between the HLA molecule and the peptides at four residues spanning the N- and C-terminal. This covers not only the anchor position sites but also their flanking sites, which have a considerable effect on peptide-HLA binding; this may also have led to the high detection rate of best-defined epitopes independent of epitope size. Together with precise HLA crystal structure information, we have also incorporated a fine calculation model for binding affinity [18], giving the DSM a high detection rate of best-defined epitopes equivalent to that of the latest binding motif-based program.

Intriguingly there was a considerable degree of discrepancy between the two methods: 21.2% of the 52 best-defined epitopes were detected as significant epitope candidates only by the DSM, while 9.6% was detected only by the HLArestrictor. Furthermore, two epitopes which ranked within the bottom five by DSM were successfully predicted as a single candidate by HLArestrictor, whereas five epitopes which were not detected by HLArestrictor, were successfully predicted as the best candidates by the DSM. This result highlights the importance of combining programs with different approaches, for example those based on peptide binding motif information and those that do not require peptide binding motif information, consistent with previous report in class II HLA peptide binding prediction model [25].

We therefore applied both models to predict optimal epitopes in HIV-1 CRF01_AE Gag and found 8 previously unreported optimal epitopes supported by both models. These potential epitopes need to be further confirmed *ex vivo* that they are true epitopes capable of stimulating T cell responses with either a ^51^Cr release assay or tetramer assay. However, since the DSM alone predicted 11 other candidates that were not predicted by the HLArestrictor, combining both models would be important to reduce the cost of such experiments. Furthermore a substantial number of OLPs were recognized using an ELISpot assay but within the peptides that induced a response, no epitope was predicted by the HLArestrictor. This DSM would save the cost of experiments by reducing 26 potential candidate peptides to five.

The ability of the DSM model to accurately predict peptides was dependent on the HLA molecule in question, and our results suggest that this is due to variations in the C-terminal binding groove. Four best-defined epitopes restricted by the alleles HLA-A*02:01 and HLA-B*57:01 ranked among the worst from the 22nd up to the 26th in our program. In HLA-A*02:01, both FK10 and AM9 coded Leucine (L) at the 2nd position of sequence, compatible with the HLA-A*02:01 binding motif at the B pocket and scored a low and therefore strongly binding U_dock score at the N-terminal site [−47.8 kcal/mol in FK10 (5th in N1-N8 terminal) and −54.4 kcal/mol in AM9 (2nd)]. However, the sequences did not match with the HLA-A*02:01 binding motif at the C-terminal which contains a Valine (V) at the F pocket, and they scored the worst U_dock scores [−14.1 kcal/mol in KF10 (8th) and −48.5 kcal/mol in AM9 (8th)]. A similarly low score at the C-terminal was also found in HLA-B*57:01-restricted KF11 [−24.5 kcal/mol (8th)] and YT9 [−23.8 kcal/mol (8th)]. The importance of the C-terminal for peptide-binding stability has been previously reported [26], and with respect to structural differences between the B and F pockets, it is generally known that the B pocket has a rather round shape while the F pocket has a deep cleft-like shape, suggesting stricter peptide binding restriction at the F pocket compared to the B pocket among HLA-A*02:01 and HLA-B*57:01. In contrast, HLA-B*27:05 and HLA-B*35:01 had none or only one variant of their binding motif at C-terminal: x[R(K)]xxxxxxx or x[R]xxxxxx[LFYRHK(MI)] in HLA-B*27:05 and x[P(AV)]xxxxxxx or x[P(AVYRD)]xxxxxx[YFMLI] in HLA-B*35:01. In these two alleles, all of the best-defined epitopes ranked within the 5th. These results strongly suggest that the diversity of peptide binding at the F pocket defines the accuracy or difficulty of epitope prediction by DSM.

Recent studies have highlighted the importance of HLA-C alleles for HIV viral control, for instance in the population-based study from Africa [27], existence of dominant HLA-C*04-restricted epitopes [28], stimulation of NK cells through HLA-C and Killer-cell Immunoglobulin-like receptors (KIRs) [29], [30], and HLA-C expression control by 35 kb upstream genotype of HLA-C allele and HIV viral control [31]. However, epitope mapping of HLA-C antigens has been held back for several reasons. Firstly, in *in vitro* studies it has been difficult to find target and effector cell combinations with singly matched HLA alleles which are not under LD association, as we found in our ^51^Cr release assay. *In silico*, in contrast to HLA-A or B alleles, epitope prediction programs against HLA-C alleles have been sparse [9]–[11]. This can be attributed to the lack of reported epitopes information from HLA-C alleles, since binding motif-based models were originally programmed based on such reported data. Furthermore, LD of HLA-C alleles, especially with HLA-B alleles, hinders the confirmation of HLA-C alleles as the restricting alleles in statistical analyses. In our previous study, among 13 HLA-C-associated epitope candidates, nine were reported with HLA-A or B alleles which were under LD association [21]. Novel DSM could contribute to epitope detection by bypassing such obstacles to epitope prediction against HLA-C alleles.

This study had several limitations. First, we could not define the threshold of the U_dock score degree itself in novel program as defined in HLArestrictor. Related with this limitation, considering the HLA polymorphism, reported epitope number, and comparison between alleles with/without original crystal structure information, further calculations will be warranted for the quality evaluation of DSM. Second, this is a computational epitope prediction model whose algorithm is solely based on the binding between the peptide and the HLA molecule. Although peptide-HLA binding is the most selective event for epitope determination [32], CTL activation is a multi-step process involving the processing of viral peptides by proteasome [22], [33], [34] and the recognition of the peptide-HLA complex by T cell receptors (TCRs) [35], both of which are not accounted for in the model.

In conclusion, we have shown here a novel *in silico* DSM which can be used for epitope mapping, and combined with a binding motif-based model, this will significantly reduce the required experimental burden for epitope identification in the development of a CTL-based vaccine for HIV.

## Supporting Information

Figure S1 Identification of HLA-B*46:01/C*01:02-restricted Gag p24_276–285_ MI10 and p24_277–285_ YI9 by a ^51^Cr release assay. ^51^Cr release assays under HLA-B*46:01/C*01:02-matched conditions were performed for each peptide. Significant % lysis was found in target cells pulsed with Gag p24_276–285_ MI10: MYSPVSILDI and p24_277–285_ YI9: YSPVSILDI.
(PPTX)

Table S1 **Predicted best-defined epitopes using the docking simulation model and a comparison with HLArestrictor.** The docking simulation model was applied to predict epitopes within 15-mer peptides spanning best-defined epitopes and compared with those predicted with the HLArestrictor. The U_dock score and their rank were calculated for each peptide in the docking simulation model, while with HLArestrictor the affinity thresholds of SB: Strong Binder, WB: Weak Binder, and CB: Combined Binder, and Non-binder were given, according to their definitions.
(XLS)

Table S2 **Epitope prediction using the docking simulation model and HLArestrictor against in vitro HLA-restricted HIV-1 CRF01_AE Gag epitope candidates.** Using previously reported HIV-1 CRF01_AE Gag epitope candidates detected by ELISpot assays and statistical analysis, epitope prediction was performed by our novel docking simulation model and HLArestrictor. Among 31 15-mer peptide and HLA associations, six best-defined epitopes and four non-best defined epitopes were included. Bold, underlined sequences indicate positive candidates in dual models. SB: Strong Binder, WB: Weak Binder, and CB: Combined Binder.
(XLSX)

## References

[1] McMichael AJ, Borrow P, Tomaras GD, Goonetilleke N, Haynes BF (2010) The immune response during acute HIV-1 infection: clues for vaccine development. Nat Rev Immunol 0: 11–23. 10.1038/nri2674 PMC3119211 20010788

[2] Mungall AJ, Palmer SA, Sims SK, Edwards CA, Ashurst JL, et al (2003) The DNA sequence and analysis of human chromosome 6. Nature 425: 805–11. 14574404 10.1038/nature02055

[3] Robinson J, Mistry K, McWilliam H, Lopez R, Parham P (2011) The IMGT/HLA Database Nucleic Acids Research. 39 (Suppl 1) D1171–6. 10.1093/nar/gkq998 PMC3013815 21071412

[4] Buonaguro L, Tornesello ML, Buonaguro FM (2007) Human immunodeficiency virus type 1 subtype distribution in the worldwide epidemic: pathogenetic and therapeutic implications. J Virol 81: 10209–19. 17634242 10.1128/JVI.00872-07 PMC2045484

[5] Pereyra F, Jia X, McLaren PJ, Telenti A, de Bakker PI, et al (2010) The major genetic determinants of HIV-1 control affect HLA class I peptide presentation. Science 330: 1551–1557. 21051598 10.1126/science.1195271 PMC3235490

[6] Draenert R, Altfeld M, Brander C, Basgoz N, Corcoran C, et al (2003) Comparison of overlapping peptide sets for detection of antiviral CD8 and CD4 T cell responses. J Immunol Methods 275: 19–29. 12667667 10.1016/s0022-1759(02)00541-0

[7] Streeck H, Frahm N, Walker BD (2009) The role of IFN-gamma Elispot assay in HIV vaccine research. Nat Protoc 4: 461–9. 19282851 10.1038/nprot.2009.7

[8] Lafuente EM, Reche PA (2009) Prediction of MHC-peptide binding: a systematic and comprehensive overview. Curr Pharm Des 15: 3209–20. 19860671 10.2174/138161209789105162

[9] Rammensee HG, Bachmann J, Emmerich NPN, Bacho OA, Stevanovic S (1999) SYFPEITHI: database for MHC ligands and peptide motifs. Immunogenetics 50: 213–9. 10602881 10.1007/s002510050595

[10] Buus S, Lauemoller SL, Worning P, Kesmir C, Frimurer T, et al (2003) Sensitive quantitative predictions of peptide-MHC binding by a ‘Query by Committee’ artificial neural network approach. Tissue Antigens 62: 378–84. 14617044 10.1034/j.1399-0039.2003.00112.x

[11] Lundegaard C, Lund O, Nielsen M (2008) Accurate approximation method for prediction of class I MHC affinities for peptides of length 8, 10 and 11 using prediction tools trained on 9mers. Bioinformatics 24: 1397–98. 18413329 10.1093/bioinformatics/btn128

[12] Erup LM, Kloverpris H, Stryhn A, Koofhethile CK, Sims S, et al (2011) HLArestrictor–a tool for patient-specific predictions of HLA restriction elements and optimal epitopes within peptides. Immunogenetics 63: 43–55. 21079948 10.1007/s00251-010-0493-5

[13] Tong JC, Tan TW, Ranganathan S (2004) Modeling the structure of bound peptide ligands to major histocompatibility complex. Protein Sci 13: 2523–32. 15322290 10.1110/ps.04631204 PMC2279999

[14] Bui HH, Schiewe AJ, von Grafenstein H, Haworth IS (2006) Structural prediction of peptides binding to MHC class I molecules. Proteins 63: 43–52. 16447245 10.1002/prot.20870

[15] Fagerberg T, Cerottini JC, Michielin O (2006) Structural prediction of peptides bound to MHC class I. J Mol Biol 356: 521–46. 16368108 10.1016/j.jmb.2005.11.059

[16] Knapp B, Omasits U, Frantal S, Schreiner W (2009) A critical crossvalidation of high throughput structural binding prediction methods for pMHC. J Comput Aided Mol Des 5: 301–7. 10.1007/s10822-009-9259-2 19194661

[17] Bordner AJ, Abagyan R (2006) Ab initio prediction of peptide-MHC binding geometry for diverse class I MHC allotypes. Proteins 63: 512–26. 16470819 10.1002/prot.20831

[18] Goto J, Kataoka R, Muta H, Hirayama N (2008) ASEDock-docking based on alpha spheres and excluded volumes. J Chem Inf Model 48: 583–90. 18278891 10.1021/ci700352q

[19] Wang J, Cieplak P, Kollman PA (2000) How well does a restrained electrostatic potential (RESP) model perform in calculating conformational energies of organic and biological molecules? J Comp Chem 21: 1049–1074.

[20] Kabsch W (1976) A solution for the best rotation to relate two sets of vectors. Acta Crystallogr Sect F Struct Biol Cryst Commun 32: 922–3.

[21] Mori M, Sriwanthana B, Wichukchinda N, Boonthimat C, Tsuchiya N, et al (2011) Unique CRF01_AE Gag CTL Epitopes Associated with Lower HIV-Viral Load and Delayed Disease Progression in a Cohort of HIV-Infected Thais. PLoS One 6: e22680. 21826201 10.1371/journal.pone.0022680 PMC3149616

[22] Yokomaku Y, Miura H, Tomiyama H, Kawana-Tachikawa A, Takiguchi M, et al (2004) Impaired processing and presentation of cytotoxic-T-lymphocyte (CTL) epitopes are major escape mechanisms from CTL immune pressure in human immunodeficiency virus type 1 infection. J Virol 78: 1324–32. 14722287 10.1128/JVI.78.3.1324-1332.2004 PMC321367

[23] Liao WW, Arthur JW (2011) Predicting peptide binding to Major Histocompatibility Complex molecules. Autoimmun Rev 10: 469–73. 21333759 10.1016/j.autrev.2011.02.003

[24] Jojic N, Reyes-Gomez M, Heckerman D, Kadie C, Schueler-Furman O (2006) Learning MHC I-peptide binding. Bioinformatics 22: e227–35. 16873476 10.1093/bioinformatics/btl255

[25] Wang P, Sidney J, Dow C, Mothe B, Sette A, et al (2008) A systematic assessment of MHC class II peptide binding predictions and evaluation of a consensus approach. PLoS Comput Biol 4: e1000048. 18389056 10.1371/journal.pcbi.1000048 PMC2267221

[26] Bouvier M, Wiley DC (1994) Importance of peptide amino and carboxyl termini to the stability of MHC class I molecules. Science 265: 398–402. 8023162 10.1126/science.8023162

[27] Leslie A, Matthews PC, Listgarten J, Carlson JM, Kadie C, et al (2010) Additive contribution of HLA class I alleles in the immune control of HIV-1 infection. J Virol 84: 9879–88. 20660184 10.1128/JVI.00320-10 PMC2937780

[28] Makadzange AT, Gillespie G, Dong T, Kiama P, Bwayo J, et al (2010) Characterization of an HLA_C-restricted CTL response in chronic HIV infection. Eur J Immunol 40: 1036–41. 20104487 10.1002/eji.200939634

[29] Jennes W, Verheyden S, Demanet C, Adjé-Touré CA, Vuylsteke B, et al (2006) Cutting edge: resistance to HIV-1 infection among African female sex workers is associated with inhibitory KIR in the absence of their HLA ligands. J Immunol 177: 6588–92. 17082569 10.4049/jimmunol.177.10.6588

[30] Ravet S, Scott-Algara D, Bonnet E, Tran HK, Tran T, et al (2007) Distinctive NK-cell receptor repertoires sustain high-level constitutive NK-cell activation in HIV-exposed uninfected individuals. Blood 109: 4296–305. 17272507 10.1182/blood-2006-08-040238

[31] Thomas R, Apps R, Qi Y, Gao X, Male V, et al (2009) HLA-C cell surface expression and control of HIV/AIDS correlate with a variant upstream of HLA-C. Nat Genet 41: 1290–4. 19935663 10.1038/ng.486 PMC2887091

[32] Jensen PE (2007) Recent advances in antigen processing and presentation. Nat Immunol 8: 1041–8. 17878914 10.1038/ni1516

[33] Tenzer S, Wee E, Burgevin A, Stewart-Jones G, Friis L, et al (2009) Antigen processing influences HIV-specific cytotoxic T lymphocyte immunodominance. Nat Immunol 10: 636–46. 19412183 10.1038/ni.1728

[34] Ranasinghe SR, Kramer HB, Wright C, Kessler BM, di Gleria K, et al (2011) The antiviral efficacy of HIV-specific CD8(+) T-cells to a conserved epitope is heavily dependent on the infecting HIV-1 isolate. PLoS Pathog 7: e1001341. 21589893 10.1371/journal.ppat.1001341 PMC3093356

[35] Dong T, Stewart-Jones G, Chen N, Easterbrook P, Xu X, et al (2004) HIV-specific cytotoxic T cells from long-term survivors select a unique T cell receptor. J Exp Med 200: 1547–57. 15596521 10.1084/jem.20032044 PMC2212004

